# Digital Health Technology Use Among Rehabilitation Professionals in China: Multi-Province Cross-Sectional Survey

**DOI:** 10.2196/90623

**Published:** 2026-04-09

**Authors:** Shuning Duan, Zi-Ru Wang, Xier Chen, Guoxin Ni

**Affiliations:** 1 Department of Rehabilitation Medicine The First Affiliated Hospital of Xiamen University School of Medicine, Xiamen University Xiamen China; 2 School of Sports Medicine Wuhan Sports University Wuhan, Hubei China

**Keywords:** digital health technologies, telehealth, rehabilitation, online survey, health technology acceptance

## Abstract

**Background:**

The rapid expansion of rehabilitation needs in China has intensified pressure on a workforce that remains unevenly distributed. Digital health technologies (DHTs) offer potential to increase service reach and efficiency. However, little is known about how rehabilitation professionals currently gather and document clinical information, nor about their readiness to integrate digital tools into routine practice within China’s rapidly digitalizing health system.

**Objective:**

This study aimed to describe how rehabilitation professionals in China collect subjective and objective clinical information, document patient data in routine practice, and assess their willingness to use DHTs in clinical settings.

**Methods:**

We conducted a multi-province observational cross-sectional survey using a culturally adapted questionnaire based on the World Health Organization Digital Health Interventions framework. The instrument assessed participant characteristics, information collection methods, documentation practices, and willingness to adopt digital functions across rehabilitation activities. Descriptive analyses and subgroup comparisons were performed on 324 complete responses from certified rehabilitation professionals. The multi-province cross-sectional online survey was conducted among licensed rehabilitation professionals in China with internet access. Participants were recruited through professional networks and social media platforms.

**Results:**

Respondents were drawn from 20 provincial-level administrative regions across China, including Fujian (n=72), Guangdong (n=77), and Shanxi (n=45), among others, with 82.7% (268/324) employed in public sector rehabilitation services. Traditional methods dominated clinical work. Face-to-face communication was used frequently for subjective assessment by 96.3% (312/324) of respondents, whereas digital channels such as email (22/324, 6.8%) and telephone (47/324, 14.5%) saw limited use. For objective information, visual observation (271/324, 83.7%) and manual measurement tools (195/324, 60.2%) remained the primary approaches, while motion capture technology (45/324, 13.8%) and wearable sensors (13/324, 4%) were rarely used. Documentation practices also relied heavily on analogue formats, with 82.1% (266/324) using handwritten notes and 60.2% (195/324) using paper templates. In contrast, willingness to adopt DHTs was consistently high, with 80.6% (261/324) of respondents indicating readiness to use digital systems for identity verification, 79.0% (256/324) for progress tracking, and 78.1% (253/324) for outcome measurement. Subgroup analyses revealed that educational level significantly influenced the adoption of advanced technologies, with master’s or doctoral degree holders reporting higher use of sensor-based assessment, motion capture, and wearable devices. In contrast, professional title and clinical specialty showed limited influence, with no significant differences observed for most digital health functions.

**Conclusions:**

Rehabilitation professionals in China demonstrate strong readiness to use DHTs, yet their routine practice remains largely paper-based and analogue. These findings provide evidence to inform implementation strategies, workforce training, and system-level planning aimed at accelerating digital transformation in rehabilitation services.

## Introduction

Rehabilitation is widely recognized as a core component of modern health systems. Global estimates indicate that more than 2 billion people live with health conditions that could benefit from rehabilitation, and this number continues to grow as populations age and chronic conditions become more common [1,2]. The role of rehabilitation in restoring function, promoting independence, and supporting healthy aging has been increasingly acknowledged in global public health priorities [3,4]. Rehabilitation is a series of interventions for individuals who are experiencing or may experience limitations in everyday functioning to help them obtain and maintain the optimum functional status [5,6]. Globally, rehabilitation services primarily address functional impairments resulting from injury, illness, or chronic conditions, encompassing disciplines such as physiotherapy, occupational therapy, and speech-language therapy [5,7,8]. Within China’s health care system, this scope aligns with the professional classifications established by the National Health Commission (NHC) and the Chinese Association of Rehabilitation Medicine, focusing on physical and functional rehabilitation rather than substance use disorder or mental health rehabilitation [9,10]. Insights into rehabilitation professionals’ current digital health practices and readiness can guide the successful implementation of these technologies in clinical settings.

Despite this growing demand, access to rehabilitation is uneven across regions. The World Health Organization (WHO) has noted that the density of rehabilitation professionals remains far below required levels in many settings, especially in low-and-middle income regions [11,12]. Previous studies have revealed that China ranked first globally in rehabilitation needs [13], yet faces severe supply shortages and regional disparities. China faces a severe workforce shortage, with only an estimated 150,000 physiotherapists serving a population of 1.4 billion. This equates to approximately 10.7 therapists per 100,000 population [14], far below the density in Australia, where physiotherapists alone account for 153.2 per 100,000 population [15,16]. Evidence indicates that China’s digital health infrastructure has expanded rapidly, with 900 internet hospitals and over 24,000 telemedicine facilities established nationwide by October 2020 [17]. However, persistent institutional barriers remain. Information silos across different levels of rehabilitation institutions impede data sharing and care coordination [18,19]. Coupled with this, gaps in the payment system exclude most digital solutions from national health insurance coverage [20,21]. In such large and complex health systems, the scale of the population and the fragmentation of service delivery further complicate efforts to expand rehabilitation capacity in a timely and coordinated manner. These challenges place additional strain on existing health care infrastructure and highlight the need for a more sustainable and scalable approach to delivering rehabilitation that can extend reach, support continuity of care, and make efficient use of limited human resources.

Digital health technologies (DHTs), as defined by the WHO, involve the use of digital and smart technologies, including artificial intelligence, the internet of things, big data, robotics, and connected devices, to improve health outcomes [22]. These technologies offer significant advantages in efficiency, scalability, and user accessibility. Evidence suggests that digital tools can support communication, monitoring, and self-management; contribute to improved outcomes; and reduce costs [23-25]. For example, in musculoskeletal rehabilitation, DHTs have emerged as transformative solutions to address the limitations of conventional care, with recent evidence demonstrating that artificial intelligence-driven motion analysis, wearable sensors, and tele-rehabilitation platforms can achieve outcomes comparable or superior to traditional approaches, alongside adherence gains of 15-40% and cost reductions of approximately 30-40% [26]. In post-trauma rehabilitation, remote digital interventions delivered via video conferencing and virtual reality (VR) have shown positive effects on physical and psychosocial outcomes for individuals with spinal cord injuries [27]. Wearable sensor technology has also gained traction in routine rehabilitation care; studies indicate that both clinicians and patients recognize its value in objectively measuring activity outside clinical settings, with most clinicians preferring continuous monitoring integrated into electronic health records to optimize workflow [28,29]. In stroke rehabilitation, home-based DHTs, including wearable devices, smartphone apps, and sensor-based solutions, have demonstrated high feasibility and usability for monitoring mobility, upper extremity function, and daily living activities [30]. Digital avatars represent another emerging modality. These approaches may be especially valuable where access to rehabilitation is limited by distance, concentration of services in urban centers, or shortages of specialized staff.

Despite the potential of DHTs to transform rehabilitation services, their development and application among rehabilitation professionals in China remain limited. Cross-disciplinary collaboration among policymakers, health care providers, technology developers, and end-users is essential to ensure that these digital solutions align with the needs of rehabilitation professionals and the patients they serve. Existing evidence suggests that digital health adoption varies across health systems and professional groups, influenced by factors such as workflow integration, technical infrastructure, and system-level support [31-33]. However, most of the existing work has focused on general practice or nursing professions, while the DHT practices of rehabilitation professionals remain comparatively underexamined. This gap is particularly notable in middle-income countries, where digital health infrastructure is expanding but the role of these tools in everyday rehabilitation practice has not been well documented [34,35]. Generating empirical data from such settings can help clarify current patterns of DHTs use and inform future implementation strategies that support appropriate and equitable integration of digital health within rehabilitation services. The findings will help guide future research in the design of DHTs as well as policymakers.

## Methods

### Study Design

A multi-province cross-sectional survey was conducted to describe the use of DHTs among rehabilitation professionals in China. The study was reported in accordance with the STROBE (Strengthening the Reporting of Observational Studies in Epidemiology) guidelines [36] (Multimedia Appendix 1). Only complete and eligible survey responses were included in the analysis.

### Instrument Development and Reliability

The survey was adapted from the clinician version of the digital health interventions (DHI) questionnaire developed by Merolli et al [37], which was created through qualitative item review of the WHO DHI classification framework [38]. The questionnaire was translated into Chinese and culturally adapted following established guidelines. The adaptation process included forward translation by 2 bilingual researchers, expert review by 5 Chinese rehabilitation specialists, back translation to ensure conceptual equivalence, cognitive interviews with 10 rehabilitation therapists, and pilot testing with 30 therapists. All adaptations focused on maintaining conceptual equivalence while ensuring clarity and relevance for rehabilitation professionals working in the Chinese health system.

The final instrument comprised four sections: (1) demographic and professional characteristics, (2) methods for obtaining subjective and objective clinical information, (3) documentation practices, and (4) willingness to use digital technologies for 32 rehabilitation-related clinical functions. All items assessing frequency and willingness used 5-point Likert scales, adapted from the approach used by Merolli et al [37]. For frequency items, responses were scored from 1 (“Never”) to 5 (“Always”). For willingness items, responses were scored from 1 (“Not at all willing”) to 5 (“very much willing”). In both cases, higher scores indicate greater frequency of use or stronger willingness to adopt. For analysis, responses of 4 or 5 were classified as “users” (for frequency items) or “willing” (for willingness items), following the approach of Merolli et al [37]. A sensitivity analysis using an alternative classification threshold (responses 3-5 defined as users and willing) confirmed that the core findings, particularly the substantial gap between willingness and actual use, remained robust regardless of the cut-off point chosen. The complete Chinese survey instrument and its English translation are provided in Multimedia Appendix 2 and Multimedia Appendix 3, respectively.

Internal consistency was assessed using Cronbach α coefficient. The results showed: subjective information collection domain (9 items): α=0.782; objective information collection domain (10 items): α=0.878; documentation practices domain (12 items): α=0.852; willingness to use digital technologies domain (32 items): α=0.986; total scale (63 items): α=0.963. All reliability coefficients exceeded the acceptable threshold of 0.70, indicating good to excellent internal consistency of the adapted questionnaire in this sample.

### Setting and Participants

This cross-sectional study was conducted between April and July 2025 among rehabilitation professionals practicing in mainland China. Eligibility was determined through 2 sequential screening questions at the beginning of the online questionnaire. The first question asked: “Have you passed the national health professional qualification examination organized by the NHC and the Ministry of Human Resources and Social Security, obtained a rehabilitation therapy qualification certificate, and are you currently providing clinical rehabilitation services in China?” (Yes or No). The remaining proceeded to the second screening question: “In the past six months, have you treated or managed an average of at least five rehabilitation patients per week?” (Yes or No). Participants represented all professional levels and practice settings, including general hospitals, rehabilitation specialty hospitals, community health centers, older adult care institutions, and sports medicine facilities.

A combination of convenience and snowball sampling was used. The survey targeted rehabilitation professionals practicing in mainland China, including physical therapists, occupational therapists, speech and language therapists, and other recognized rehabilitation therapy practitioners. In China, professional roles in rehabilitation can sometimes overlap, with practitioners potentially assuming responsibilities across multiple specialty areas. To address this complexity and ensure accurate classification of participants’ primary professional focus, we adopted a classification framework based on core clinical responsibilities. This framework was informed by the professional definitions of World Physiotherapy and the World Federation of Occupational Therapists [39,40], and adapted to align with the specialty classifications of the Chinese Association of Rehabilitation Medicine [41,42] Specifically, respondents were asked to select their primary practice area from 9 predefined categories (eg, neurological rehabilitation, musculoskeletal rehabilitation, and pediatric rehabilitation) and to describe their routine responsibilities in an open-ended question. Based on their responses, we classified participants into the most appropriate professional category for analysis.

### Recruitment and Data Collection

Data were collected between April and July 2025 using an anonymous online survey hosted on Questionnaire Star (Changsha Ranxing Science and Technology Co, Ltd), a widely used Chinese survey platform. Recruitment occurred through multiple channels, including professional networks, departmental distribution within hospitals, social media groups for rehabilitation therapists, and peer referral. Potential participants accessed the survey through an open link. The front page of the survey clearly stated the research purpose, assured anonymity, and emphasized voluntary participation; proceeding to the questionnaire implied informed consent. After screening questions confirmed eligibility, participants were able to proceed to the full survey. Duplicate submissions were limited using single-device submission control, and no incentives were offered.

### Quality Control

To ensure data quality, we implemented multilayered quality control measures throughout the recruitment process. As illustrated in the participant flowchart, of the 393 initial responses received, 52 were excluded for not meeting eligibility criteria (lack of national qualification or not in clinical practice), and an additional 17 were excluded for not meeting the minimum caseload requirement (fewer than 5 patients per week). Following eligibility screening, responses were further scrutinized through automated checks, including IP address to detect duplicate submissions, and manual review of completion times and response patterns. After these quality control procedures, 324 valid responses were retained for final analysis.

### Data Analysis

Data from completed surveys were analyzed descriptively using SPSS (version 27; IBM Corp). Categorical variables were summarized using frequencies and percentages. For Likert-scale items, responses of always or frequently were classified as users, and responses of very much willing or quite a bit were classified as willing, following the approach used by Merolli et al [37]. Descriptive secondary comparisons were conducted across demographic and professional subgroups to explore variation in DHT use and willingness. For all subgroup comparisons, Kruskal-Wallis tests were used due to the non-normal distribution of the data. Post-hoc pairwise comparisons were conducted using Dunn test with Bonferroni correction to control for Type I error. Effect sizes (ε²) were calculated manually using the formula ε² = (H – k + 1) / (N – k), where k represents the number of groups and N the total sample size. Effect sizes were interpreted as small (0.01), medium (0.06), or large (0.14). Open-ended responses were reviewed using a directed qualitative content approach to summarize commonly reported barriers and facilitators.

Missing data were handled through listwise deletion based on the study’s eligibility criteria. After applying the predefined exclusion standards (qualification, practice status, and minimum caseload requirements), 324 complete and valid responses were retained for final analysis, with no missing values in any key variables.

### Ethical Considerations

Ethics approval for this study was granted by the ethics committee of the First Affiliated Hospital of Xiamen University (approval XMFHIIT-2025SL023) in accordance with institutional guidelines. Informed consent was obtained from all the participants, and data were anonymized to ensure privacy and confidentiality. No compensation was provided for participation.

## Results

### Overview

**Figure 1:**
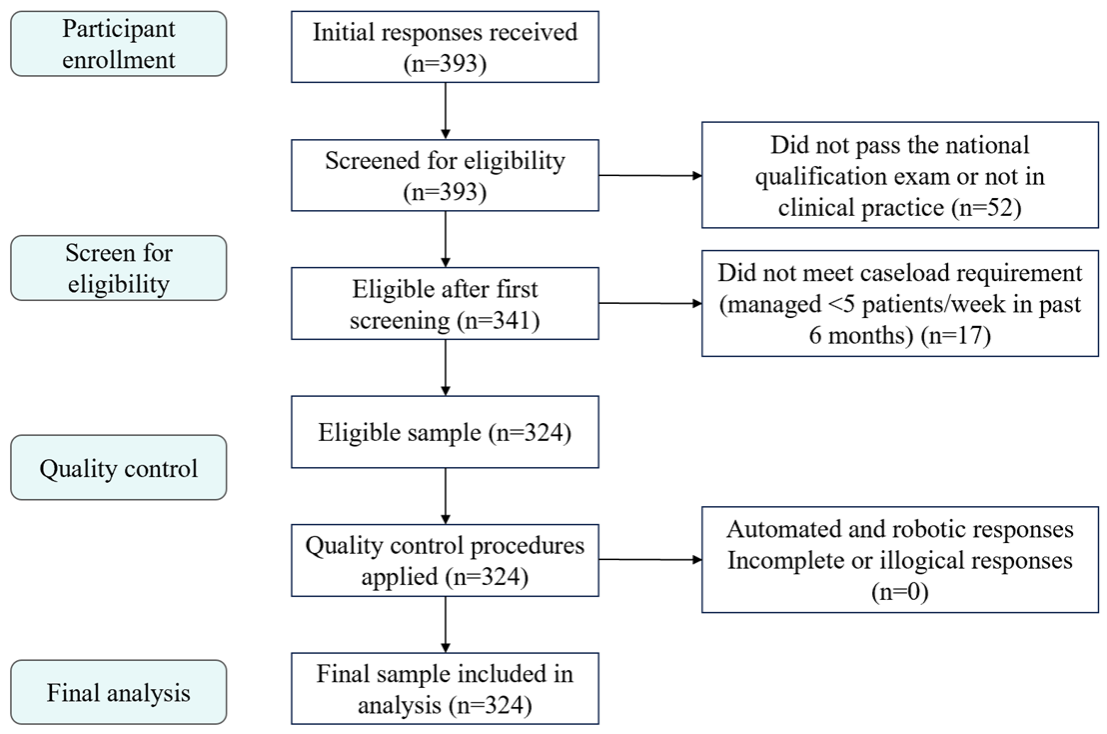
Recruitment flowchart for this study.

This survey collected a total of 393 questionnaires from 20 provinces across China, of which 324 met the eligibility criteria. The exclusion criterion was failure to pass the NHC qualification examination, not currently practicing in mainland China, or having treated or managed an average of fewer than 5 rehabilitation patients per week over the 6 months preceding the survey. Upon receipt of the research participant invitation letter and relevant study information, voluntary participation in the survey constituted informed consent. Figure 1 presents the detailed participant flow diagram for this study.

### Participant Characteristics

**Figure 2:**
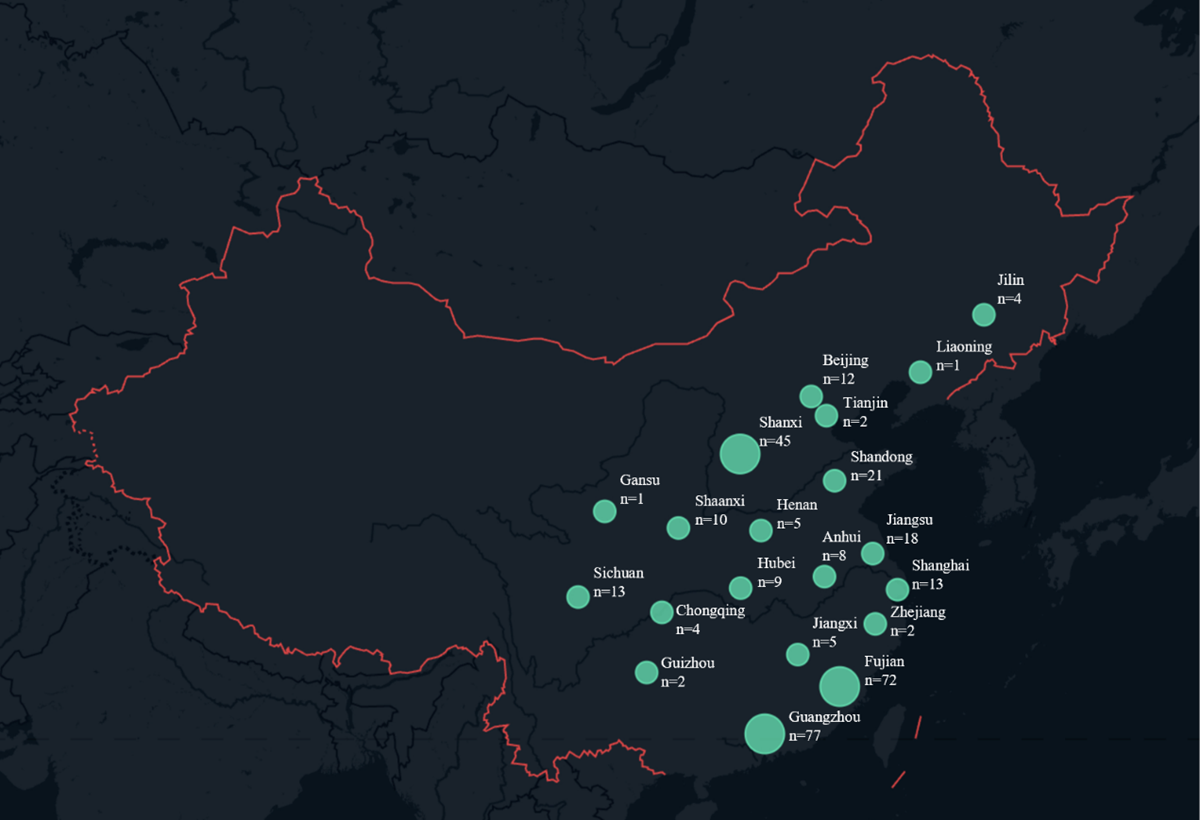
Geographic distribution of survey respondents (N=324).

A total of 324 certified rehabilitation professionals were included in the analysis. Most were aged 20 to 29 years (209/324, 64.5%) and female (173/324, 53.4%). Nearly half held the title rehabilitation therapist (158/324, 48.8%), with 26.9% (87/324) classified as senior rehabilitation therapists. Clinical experience was most commonly 2-5 years (157/324, 48.5%), and the majority held a bachelor’s degree (209/324, 64.5%). Most participants worked within public-sector general hospital rehabilitation departments (268/324, 82.7%), and internet access was near-universal, primarily via mobile phone (322/324, 99.4%). Table 1 summarizes demographic characteristics. The geographic distribution of respondents is illustrated in Figure 2, with the highest proportions from Guangdong (77/324, 23.8%), Fujian (72/324, 22.2%), and Shanxi (45/324, 13.9%).

**Table 1:** Participant characteristics (N=324).

Characteristics		Participants, n (%)
**Sex**		
	Male	151 (46.6)
	Female	173 (53.4)
**Age (years)**		
	20-29	209 (64.5)
	30-39	96 (29.6)
	40-49	18 (5.6)
	50-59	1 (0.3)
**Professional title**		
	Rehabilitation assistant	60 (18.5)
	Rehabilitation therapist	158 (48.8)
	Senior rehabilitation therapist	87 (26.9)
	Associate chief rehabilitation therapist	16 (4.9)
	Chief rehabilitation therapist	3 (0.9)
**Years of clinical experience**		
	<2 years	52 (16)
	2-5 year	157 (48.5)
	6-10 years	68 (21)
	11-15 years	27 (8.3)
	16-20 years	13 (4)
	>20 years	7 (2.2)
**Highest education level**		
	Associate degree	44 (13.6)
	Bachelor’s degree	209 (64.5)
	Master’s degree	62 (19.1)
	Doctoral degree	9 (2.8)
**Work sector (institutional ownership)**		
	Private institution	55 (17)
	Public institution	253 (78.1)
	Both public and private	13 (4)
	Other	3 (0.9)
**Primary work setting (institution type)**		
	General hospital rehabilitation department	268 (82.7)
	Rehabilitation specialty hospital	28 (8.6)
	Community health center	6 (1.9)
	Elder care institution	12 (3.7)
	Sports team or sports medicine center	1 (0.3)
	Other	9 (2.8)
**Weekly clinical hours**		
	<5	14 (4.3)
	6-10	64 (19.8)
	11-20	11 (3.4)
	21-30	20 (6.2)
	31-40	128 (39.5)
	>40	87 (26.9)
**Primary clinical area**		
	Neurological rehabilitation	138 (42.6)
	Musculoskeletal rehabilitation	69 (21.3)
	Cardiorespiratory rehabilitation	11 (3.4)
	Pediatric rehabilitation	35 (10.8)
	Geriatric rehabilitation	26 (8)
	Trauma rehabilitation	2 (0.6)
	Mental health rehabilitation	3 (0.9)
	Speech, swallowing, and hearing rehabilitation	29 (9)
	Occupational rehabilitation	2 (0.6)
	Others	9 (2.8)
**Province**		
	Fujian	72 (22.2)
	Guizhou	2 (0.6)
	Guangdong	77 (23)
	Shanxi	45 (13.9)
	Beijing	12 (3.7)
	Henan	5 (1.5)
	Sichuan	13 (4)
	Jilin	4 (1.2)
	Jiangxi	5 (1.5)
	Shaanxi	10 (3.1)
	Gansu	1 (0.3)
	Shandong	21 (6.5)
	Chongqing	4 (1.2)
	Tianjin	2 (0.6)
	Hubei	9 (2.8)
	Shanghai	13 (4)
	Liaoning	1 (0.3)
	Anhui	8 (2.5)
	Zhejiang	2 (0.6)
	Jiangsu	18 (5.5)

### Collection of Subjective Clinical Information

**Figure 3:**
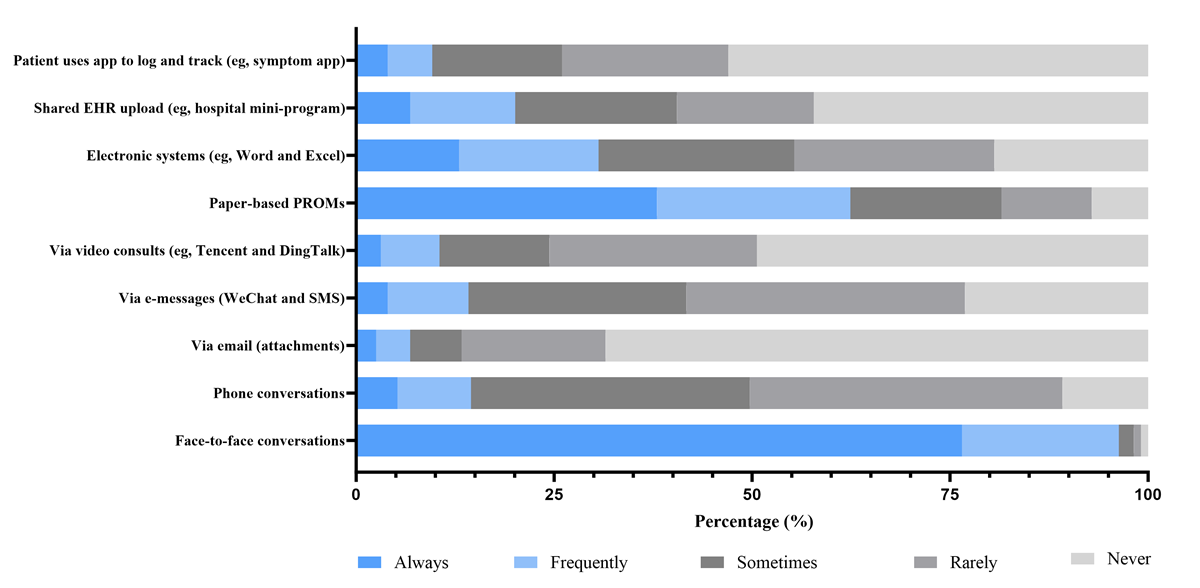
Frequency of methods and tools used to collect subjective clinical information. EHR: electronic health record; PROM: patient-reported outcome measure.

Figure 3 illustrates a marked reliance on in-person communication, with 96.3% (312/324) reporting frequent or constant use of face-to-face discussions, far exceeding any other method. Digital communication channels were rarely incorporated into routine assessment. Only 14.2% (46/324) used e-messages, 14.5% (47/324) used phone calls, and 6.8% (22/324) used email with any regularity. Paper-based patient-reported outcome measures (PROMs) were far more established (202/324, 62.4% frequent use) than electronic formats (99/324, 30.6%), indicating that digital patient-reported data capture has not yet been scaled into routine practice.

### Collection of Objective Clinical Information

**Figure 4:**
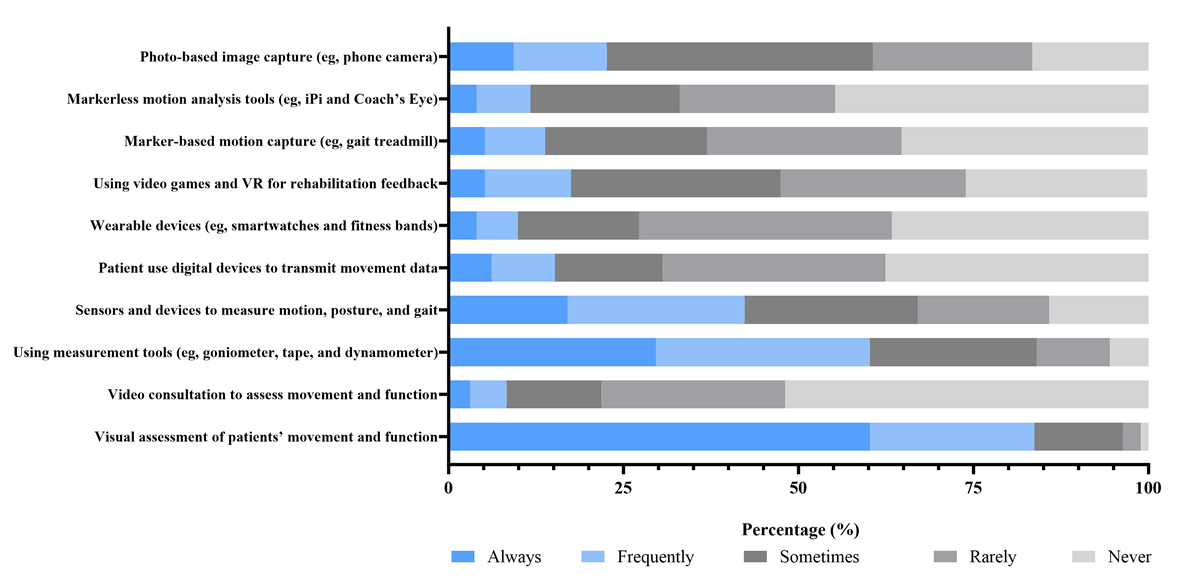
Frequency of methods and tools used to collect objective clinical information. VR: virtual reality.

Figure 4 demonstrates similar dominance of traditional approaches for objective assessment. Visual observation was the primary method (271/324, 83.7% frequently or always), while manual measurement tools such as goniometers and dynamometers were also widely used (195/324, 60.2%). In contrast, digital motion-capture technologies, VR-based tools, wearable sensors, and patient-generated device data showed limited integration, with frequent use ranging between 4 to 14% across categories. More than 70% (227/324) rarely or never accessed wearable-generated metrics, and video-based assessment remained infrequent. These patterns reflect early-stage digital adoption rather than widespread integration of technology-supported movement analysis.

### Documentation of Clinical Assessment Information

**Figure 5:**
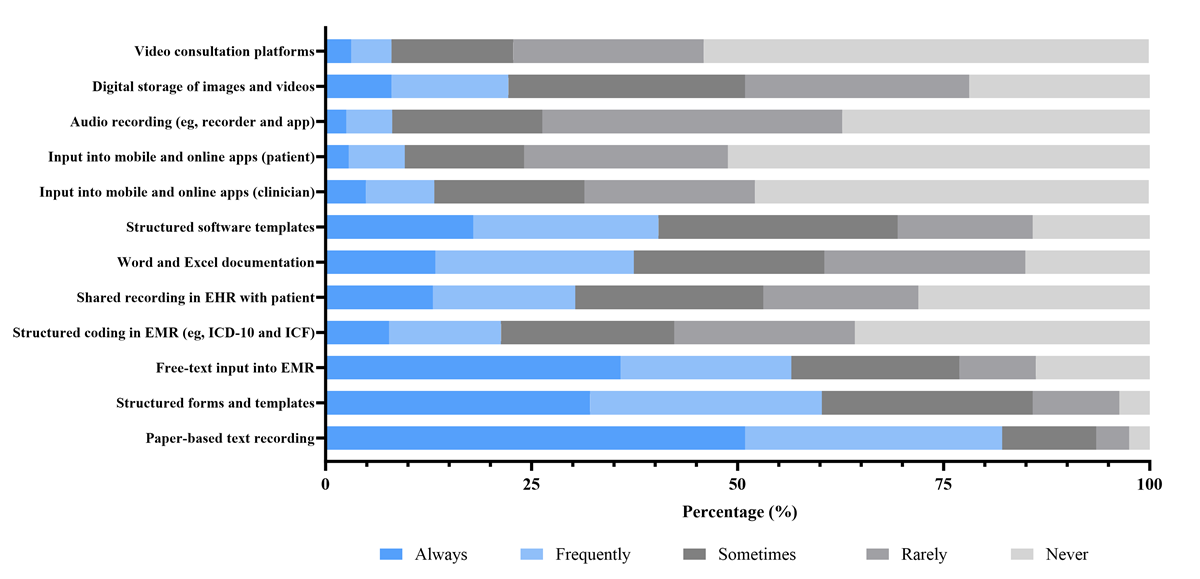
Frequency of methods and tools used to document clinical assessment information. EHR: electronic health record; EMR: electronic medical record; ICD-10: International Statistical Classification of Diseases, Tenth Revision; ICF: International Classification of Functioning, Disability and Health.

As displayed in Figure 5, handwritten free-text notes (266/324, 82.1%) and structured paper templates (195/324, 60.2%) formed the dominant documentation format. Unstructured EMR entry was more commonly used than structured coding (183/324, 56.5% vs 69/324, 21.3%), suggesting fragmented rather than standardized digital documentation. Microsoft Word or Microsoft Excel documentation was used occasionally (121/324, 37.4% combined frequent or occasional use), but mobile app–based entries, patient-entered digital notes, audio files, and video and image storage remained minimal, with most respondents reporting rare or no use. The predominance of paper systems suggests a slow transition toward digitalized information environments.

### Willingness to Use Digital Technologies

**Figure 6:**
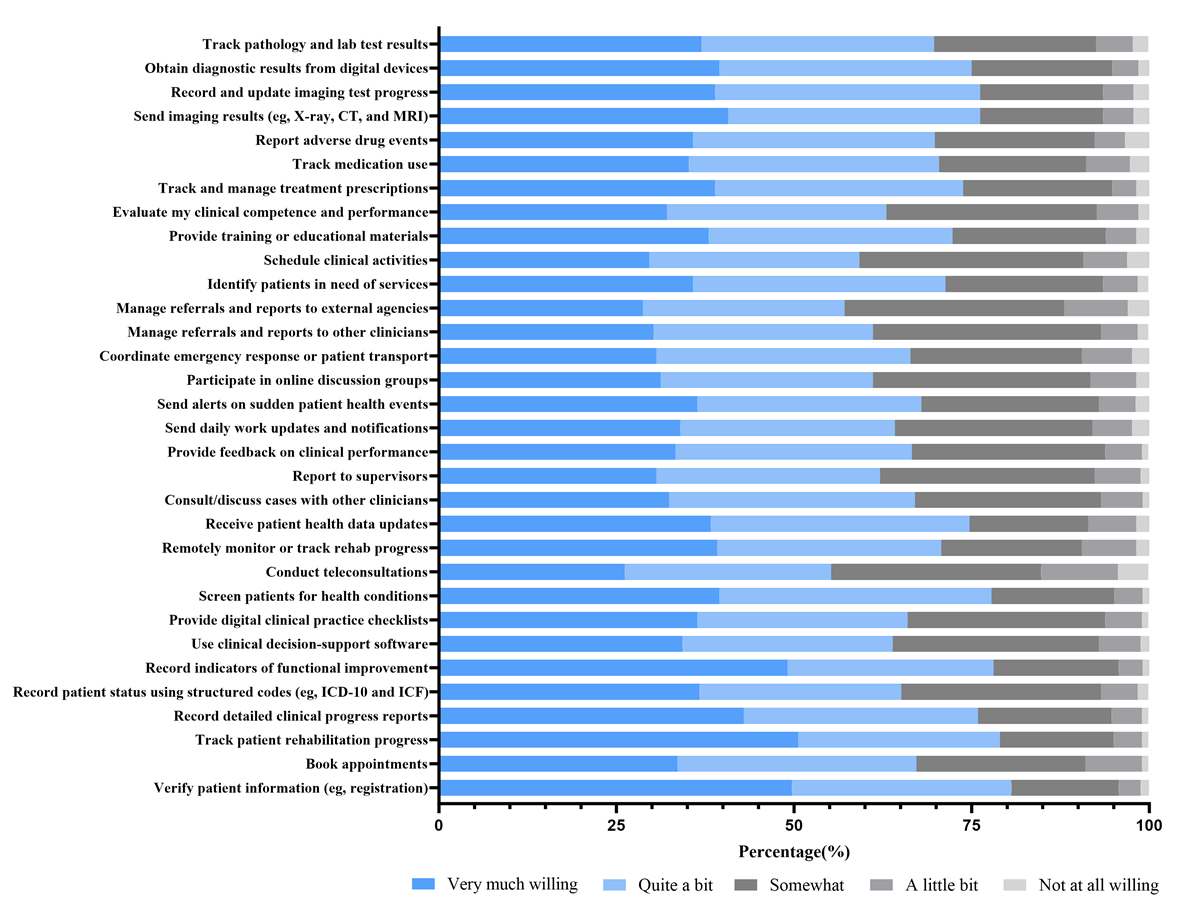
Willingness to use digital technologies for rehabilitation-related clinical activities. CT: computed tomography; ICD-10: International Statistical Classification of Diseases, Tenth Revision; ICF: International Classification of Functioning, Disability and Health; MRI: magnetic resonance imaging.

Figure 6 shows generally high willingness across nearly all digital functions assessed. High proportions of respondents were willing to use digital systems for identity verification (261/324, 80.6%), progress tracking (256/324, 79.0%), and outcome measurement (253/324, 78.1%), all exceeding 75%. Willingness also exceeded 70% for accessing imaging (235/324, 72.5%), receiving clinical alerts (231/324, 71.3%), and recording treatment changes (228/324, 70.4%). Lower but still favorable levels were observed for teleconsultation (179/324, 55.2%) and digital referral coordination (185/324, 57.1%). The overall mean willingness score across these digital functions was 4.02, with moderate SD values (0.90-1.11), indicating consistent support for digital adoption within the cohort. Overall, expressed willingness was consistently higher than reported current use across digital domains, indicating a gap between readiness and practice.

### Subgroup Analyses by Demographic and Clinical Specialty

Subgroup analyses showed that educational level significantly influenced the adoption of advanced technologies, while professional title and clinical specialty had limited effects. Full details of the Kruskal-Wallis test results, including H values, *P* values, effect sizes, and post-hoc comparisons, are provided in Multimedia Appendix 4.

Findings revealed significant differences across educational levels for several outcomes. Sensor-based assessment (H=24.801, *P*<.001, ε²=0.068), marker-based motion capture (H=23.052, *P*<.001, ε²=0.063), wearable device data (H=13.695, *P*=.003, ε²=0.033), electronic systems (H=10.823, *P*=.01, ε²=0.024), and unstructured free-text EMR entry (H=12.214, *P*=.007, ε²=0.029) all varied significantly, with post-hoc comparisons showing that therapists with master’s or doctoral degrees consistently reported higher use than those with associate or bachelor’s degrees. By professional title, significant differences were found for sensor-based assessment (H=18.904, *P*<.001, ε²=0.047), wearable device data (H=13.142, *P*=.01, ε²=0.029), unstructured free-text EMR entry (H=11.851, *P*=.02, ε²=0.025), and teleconsultation willingness (H=11.802, *P*=.02, ε²=0.024). Post-hoc analyses indicated that rehabilitation assistants and therapists generally reported lower use and willingness than senior or associate chief therapists. Across clinical specialties, limited differences were observed: sensor-based assessment (H=25.507, *P*=.002, ε²=0.052) and face-to-face communication (H=25.134, *P*=.003, ε²=0.051) showed significant variation, with speech and language therapists using sensors more frequently than neurological or musculoskeletal therapists, and mental health therapists using face-to-face communication less frequently than several other specialties. Unstructured free-text EMR entry (H=18.112, *P*=.03, ε²=0.029) and willingness to send imaging results (H=18.075, *P*=.03, ε²=0.029) reached overall significance, but no pairwise comparisons remained significant after correction. No other outcomes differed significantly across groups (all *P*>.05).

### Summary of Open-Ended Responses

Open-ended responses were reviewed to provide additional context to the quantitative findings. Commonly reported barriers to digital health use included limited interoperability between information systems, continued reliance on paper-based workflows, insufficient institutional support, and time constraints in routine clinical practice. Respondents also highlighted perceived benefits of digital technologies, particularly in improving efficiency, data continuity, and long-term patient monitoring. These responses did not introduce themes that contradicted the quantitative results.

## Discussion

### Principal Findings

This cross-sectional survey provides new evidence on how rehabilitation professionals in China use DHTs in routine practice. Given that implementation of DHIs represents a cultural transformation of traditional health care [43], understanding the confidence and attitudes of health care professionals regarding the use of DHTs is a growing priority [44]. In this sample, in-person communication (312/324, 96.3%) and paper-based documentation (266/324, 82.1%) were the dominant methods for obtaining clinical information. In contrast, the adoption of digital tools remained limited across all domains. For instance, the frequent use of email (22/324, 6.8%), patient-initiated app-based data sharing (32/324, 9.6%), and video consultations for objective assessment (27/324, 8.3%) each fell below 10%. Despite this limited uptake, respondents expressed strong willingness to incorporate digital technologies into their clinical work, with more than 70% reporting positive attitudes toward most digital functions. This high readiness stands in stark contrast to the low levels of actual use, revealing a substantial willingness-practice gap. For example, although 79% (256/324) of respondents were willing to digitally track patient rehabilitation progress, only 30.6% (99/324) frequently used electronic systems to collect subjective information. Similarly, while 55.2% (179/324) expressed willingness to conduct teleconsultations, the actual frequent use of video-based objective assessment was only 8.3% (27/324). These findings underscore a significant opportunity to advance the digital transformation of rehabilitation services.

Previous research in other regions shows both alignment and divergence with our findings. An Australian survey using the original version of this instrument [37] reported widespread use of EMR-based free-text documentation and strong willingness to adopt digital tools for tasks such as scheduling, imaging tracking, and teleconsultation, yet therapists still relied predominantly on face-to-face communication for subjective assessment and visual observation for objective evaluation. In comparison, Chinese therapists showed similar dependence on direct communication but demonstrated broader, more experimental uptake of digital methods, including photo, video, and sensor-based assessment, while maintaining heavier reliance on paper-based records. Although willingness to use digital technologies was high in both contexts, the emphasis differed: Australian clinicians prioritized tools that streamline communication and workflow, whereas Chinese therapists expressed greater interest in outcome monitoring, identity verification, and progress tracking rather than patient-facing teleconsultation. These differences likely reflect cultural preferences for relationship-centered interaction, earlier digitization of health records in Australia, and variability in hospital information systems across Chinese provinces [45].

Findings from other regions further contextualize these patterns. An Italian survey reported high availability of digital equipment but low levels of perceived integration and knowledge, alongside marked regional disparities in resources and experience [46]. Similarly, Norwegian physiotherapists identified telephone consultation as the most commonly used digital communication modality, with limited uptake of video consultation, gaming, or VR technologies [47]. Studies from the Middle East, North Africa, and other middle-income settings likewise describe early-stage digital adoption within rehabilitation systems, characterized by strong enthusiasm but persistent barriers related to software availability, interoperability, digital literacy, and organizational readiness [48-50]. Collectively, this body of evidence suggests that globally, clinician enthusiasm for digital rehabilitation often outpaces the degree of practical implementation [51-53]. Our study extends this literature by providing empirical data from a rapidly digitizing health system, highlighting both shared challenges and substantial untapped potential.

The substantial willingness-practice gap observed in our study is consistent with recent findings from other rehabilitation settings in China. A survey of orthopedic rehabilitation medical staff found that while support for digital therapeutics was high, only 21% had actually used them, with concerns about privacy, interpersonal relationships, and time constraints limiting adoption [54]. Similarly, a study of autism spectrum disorder digital health services across 2 Chinese provinces reported that despite high demand among rehabilitation therapists (90.0%), actual use remained critically low (21.6%) [55]. Together, these findings suggest that the gap between readiness and implementation is not unique to our sample but reflects a broader systemic challenge across diverse rehabilitation disciplines in China. Our results also suggest that the strong demand for digital health training (234/324, 72.3%) highlights a critical leverage point. This finding aligns with previous research indicating that the absence of systematic, formal education on digital technologies limits the readiness of current and future health professionals for digital transformation [56]. It suggests that rehabilitation professionals are not only ready to adopt digital tools but are also actively seeking opportunities to develop the skills required for their effective implementation. Bridging this educational gap could accelerate the translation of high willingness into tangible clinical practice by integrating digital health competencies into entry-level curricula and establishing continuing professional development programs.

This study also examined the influence of educational background, professional title, and clinical specialty on digital health use patterns. Subgroup analyses revealed that educational level was the most significant factor influencing the adoption of advanced technologies, with therapists holding master’s or doctoral degrees reporting significantly higher use of sensor-based assessment, motion capture technology, and wearable devices compared to those with associate or bachelor’s degrees. In contrast, the influence of professional title was limited; although significant differences emerged in a few indicators, most post-hoc comparisons were nonsignificant after adjustment, and professional title had no effect on the vast majority of digital health functions. Similarly, differences across clinical specialties were minimal. The absence of differences across most domains suggests that readiness for digital rehabilitation is broadly shared across specialties, rather than being driven by discipline-specific factors. This pattern points toward the influence of system-level conditions rather than professional background alone in shaping digital health adoption in rehabilitation practice. Insights from open-ended responses further support the interpretation that barriers to digital rehabilitation are primarily structural and organizational rather than related to clinician willingness. The implications of these findings are considerable. Given China’s large population, rising burden of disabling conditions, and uneven geographic distribution of rehabilitation resources, the high level of willingness among therapists represents a critical opportunity [57-59].

Scaling up digital health could help extend services to underserved regions, improve continuity of care, reduce travel burden for patients, and partially mitigate workforce shortages [60,61]. Evidence from digital health implementation research indicates that the development of supportive policy environments, including reimbursement arrangements, data governance structures, and standardized documentation frameworks, can enable more durable adoption of technology in rehabilitation services [62-64]. Research in digital health education also highlights the value of structured training, with multiple evaluations reporting that integration of telehealth and digital assessment skills into both preservice and continuing professional programs improves clinician confidence and routine application [65,66]. Evidence from programs in musculoskeletal rehabilitation further suggests that remote teleconsultations can have the same effect as in-person physio consultations and can maintain clinical outcomes while expanding reach to populations with limited physical access [24]. Meanwhile, secondary analyses in our study revealed a positive trend in willingness to engage in teleconsultation across increasing professional titles, with higher-ranking therapists reporting greater willingness. This finding underscores the need for tiered digital training strategies tailored to rehabilitation professionals at different career stages, with particular attention to supporting lower- and mid-level practitioners. In addition, studies from low-resource regions show that software availability, device access, and internet reliability strongly influence digital function, with rural and underresourced settings experiencing the greatest barriers [67,68].

Taken together, this study demonstrates that limited use of DHTs in rehabilitation practice should not be interpreted as a lack of clinician readiness. Instead, our findings indicate a widespread willingness to engage with digital rehabilitation across specialties and professional levels, alongside persistent structural and organizational constraints that limit implementation. By providing evidence from China, this study contributes to a growing body of international research showing that the digital transformation of rehabilitation is primarily shaped by system-level conditions rather than individual clinician attitudes. These insights underscore the need for implementation strategies that move beyond profession-specific solutions and address the broader institutional, infrastructural, and policy environments in which rehabilitation services are delivered.

### Limitations

This study has several limitations. This study has several limitations. Due to open-link recruitment, the response rate could not be calculated, precluding assessment of nonresponse bias. The online survey mode inherently introduced selection bias by systematically excluding professionals with limited internet access or digital skills, and participation itself implied a certain level of comfort with digital technologies. This self-selection mechanism likely overestimates the overall population’s willingness and actual use levels, particularly for those in remote areas or primary care settings. Geographic distribution bias further limits generalizability, as respondents were predominantly from several provinces, with underrepresentation from central and western regions and primary care institutions. Additionally, all data were based on self-reports, which may be subject to social desirability and recall biases. The cross-sectional design captures technology use at a single time point and cannot reveal dynamic changes in adoption or causal pathways, underscoring the need for longitudinal studies.

### Conclusions

In conclusion, clinical practice in China continues to rely primarily on traditional in-person and paper-based methods, yet clinicians express clear readiness to adopt digital health. This readiness indicates significant potential for targeted policy actions, education programs, and implementation planning. Addressing practical and structural barriers could accelerate digital transformation in rehabilitation and support improved access and more sustainable delivery of rehabilitation services.

## Data Availability

The data that support the findings of this study are available from the corresponding author upon reasonable request.

## Appendix

Multimedia Appendix 1 STROBE checklist.

Multimedia Appendix 2 Survey instrument for rehabilitation therapists on digital health applications (Chinese version).

Multimedia Appendix 3 Survey instrument for rehabilitation therapists on digital health applications (Chinese back-translation to English for review).

Multimedia Appendix 4 Subgroup comparisons of digital health technology use by educational level, professional title, and clinical specialty.

## Abbreviations

DHI: digital health interventions
DHT: digital health technology
NHC: National Health Commission
STROBE: Strengthening the Reporting of Observational Studies in Epidemiology
VR: virtual reality
WHO: World Health Organization
