# Supplementary material for: Digital Health Technology Use Among Rehabilitation Professionals in China: Multi-Province Cross-Sectional Survey
Source: J Med Internet Res. 2026 Apr 9;28:e90623. doi: 10.2196/90623 (PMC13107106; doi:10.2196/90623)
Supplement: Multimedia Appendix 2 [file jmir_v28i1e90623_app2.docx]

**Multimedia Appendix 2.** **Survey Instrument for Rehabilitation Therapists on Digital Health Applications (Chinese Version)**

**请确认您有资格继续进行调查**

1. 您是否通过了国家卫生健康委员会（NHC）和人力资源和社会保障部（MOHRSS）组织的全国卫生专业技术资格考试，并获得康复治疗资格证书，并且目前正在中国提供康复治疗临床服务？

□ 是

□ 否

2. 过去的6个月里，您是否平均每周至少治疗或管理5名康复患者?

□ 是

□ 否

**第1部分 – 基本信息**

我们想问一些关于您个人及您临床经验的简要问题。

3. 请问您的职称等级是什么？

□ 康复治疗士

□ 康复治疗师

□ 主管康复治疗师

□ 副主任康复治疗师

□ 主任康复治疗师

□ 其他（请注明）：________________________________________________

4. 您是如何得知这次调查的？

□ 电子邮件

□ 社交媒体（包括朋友圈）

□ 大学/医院网站

□ 海报/传单

□ 通讯

□ 同事或同行推荐

□ 其他（请指明）：________________________________________________

5. 您使用什么设备上网？（请选择所有适用的选项）

□ 手机

□ 平板电脑（如iPad等）

□ 台式电脑

□ 笔记本电脑

□ 智能电视

□ 游戏机

□ 智能手表（如 Apple Watch、华为手表、小米手环等）

□ 电子书阅读器（如掌阅I Reader、小米多看）

□ 智能家居助手（如小米小爱同学、天猫精灵）

□ 其他（请注明）：________________________________________________

6. 您上网/访问互联网的频率是多少？

□ 每小时

□ 每日

□ 每周

□ 每月

□ 少于每月

7. 您的性别是什么？

□ 男

□ 女

8. 请告诉我们您的年龄范围？

□ < 20

□ 20-29

□ 30-39

□ 40-49

□ 50-59

□ 60-69

□ 70+

9. 您拥有多少年的临床经验（自获得康复治疗资格证以来）？

□ <2年

□ 2-5

□ 6-10

□ 11-15

□ 16-20

□ >20

10. 您的最高学历是什么

□ 中专

□ 大专

□ 本科

□ 硕士研究生

□ 博士研究生

□ 其他（请指明）：________________________________________________

11. 您在什么机构/部门从事康复治疗工作？

□ 私立机构

□ 公立机构

□ 私人和公立机构均有

□ 其他（请指明）：________________________________________________

12. 您主要在哪种类型的医疗机构从事康复治疗工作？（请选择您的主要工作单位）

□ 综合医院康复科

□ 康复专科医院

□ 社区卫生服务中心

□ 养老机构

□ 残疾人康复中心

□ 运动队/运动医学中心

□ 其他（请注明）：________________________________________________

13. 您平均每周有多少小时从事康复治疗临床实践？

□ <5

□ 6-10

□ 11-20

□ 21-30

□ 31-40

□ >40

14. 您主要工作地点的所在城市

________________________________________________________________

15. 您**最主要的**康复治疗实践领域是什么？（单选）

□ 神经康复

□ 肌肉骨骼康复

□ 心肺康复

□ 儿童康复

□ 老年康复

□ 创伤康复

□ 肿瘤康复

□ 运动损伤康复

□ 精神心理康复

□ 言语、吞咽与听力康复

□ **职业康复**

□ 其他（请注明）：________________________________________________

16. 以下哪项最符合您的职业职责？

□ **物理治疗师：**我通过运动、手法或仪器治疗患者的功能障碍，例如术后康复或中风后的功能恢复。

□ **运动康复师：**我专注于设计运动方案，帮助患者预防运动损伤或提升运动能力，例如运动损伤恢复期的训练。

□ **作业治疗师：**我帮助患者恢复日常生活能力，例如重新学习穿衣、进食或职业技能。

□ **言语治疗师：**我评估并改善患者的语言、吞咽功能障碍。

□ **心肺康复治疗师**：我专注于为心肺疾病患者设计和实施康复计划，以增强患者心肺功能和整体健康水平。

□ **假肢与矫形器师：**我通过调整假肢或矫形器，改善患者的生活质量和行动能力。

□ **儿童康复治疗师：**我为患有语言障碍、发育迟缓、自闭症以及脑瘫等儿童进行评估，并进行个性化治疗，改善并促进患儿的功能。

□ 其他（请注明）：________________________________________________

**第2部分：评估方式**

以下问题涉及您在康复治疗过程中如何主观和客观地**收集**和**记录**患者信息

**以下部分关于主观信息（即患者个人汇报的信息）收集：**

17. 请在“**从不**”到“**总是**”中选择最佳答案，以表明

您有**多频繁地**使用所列的每种方法或工具**从需要康复的患者**那里获取**主观临床信息**

| **问题** | **从不使用** | **很少使用** | **有时使用** | **经常使用** | **总是使用** |
| --- | --- | --- | --- | --- | --- |
| 面对面交谈 |  |  |  |  |  |
| 电话交谈 |  |  |  |  |  |
| 通过电子邮件（包括发送附件） |  |  |  |  |  |
| 通过电子消息（包括短信、彩信或其他社交媒体） |  |  |  |  |  |
| 通过视频会议/远程咨询（例如腾讯视频、钉钉等） |  |  |  |  |  |
| 使用纸质问卷或由患者填写的结果测量表 |  |  |  |  |  |
| 使用电子系统采集患者填写的主观信息（例如Word、Excel等） |  |  |  |  |  |
| 我的患者将信息上传到我可以访问的共享电子健康记录中（例如挂号时患者在医院小程序里填写的个人信息） |  |  |  |  |  |
| 我的患者使用APP记录/跟踪他们的病情，我可以访问/查看这些信息（例如用于症状监督或运动处方的专用APP） |  |  |  |  |  |

18. 请说明您用来收集患者**主观**信息任何其他方法或工具（如果有请注明，如果没有请填“无”）：

________________________________________________________________

**以下部分关于客观信息（外部测量或检验的信息）收集的：**

19. 请选择从“**从不**”到“**总是**”的最佳答案，以表示

您有**多频繁地**使用所列的每种方法或工具**从患者那里**获取**客观临床信息**

| 问题 | 从不使用 | 很少使用 | 有时使用 | 经常使用 | 总是使用 |
| --- | --- | --- | --- | --- | --- |
| 目测评估患者的运动和功能 |  |  |  |  |  |
| 通过视频会诊（如腾讯会议或钉钉）评估患者的运动和功能 |  |  |  |  |  |
| 使用测量设备（例如量角器、卷尺、握力计、压力袖带等） |  |  |  |  |  |
| 使用传感器或设备测量运动、姿势、平衡、步态、肌肉活动等（例平衡测试仪、步态分析系统等） |  |  |  |  |  |
| 我的患者利用临床传感器或应用程序来监测他们的运动表现、姿势、平衡、步态以及肌肉活动。即便在他们不在医疗中心/康复机构的情况下，我也能远程访问这些数据。（例如，用于评估姿势的可穿戴传感器等） |  |  |  |  |  |
| 使用患者所佩戴的个人活动监测设备（例如苹果手表、华为手表、小米手环等）所提供的信息 |  |  |  |  |  |
| 使用电子游戏和虚拟现实技术（例如：HTC VIVE、Oculus Rift、索尼PlayStation VR等）作为康复治疗的一部分，可以获得关于患者的表现/进展的信息 |  |  |  |  |  |
| 使用基于视频的运动捕捉技术分析患者功能水平，这涉及到在患者身上粘贴标记点（例如使用步态分析跑台）以追踪和记录运动细节。 |  |  |  |  |  |
| 使用无标记技术运动分析工具(例如, iPi Motion Capture和Coach's Eye等)进行评估 |  |  |  |  |  |
| 使用基于照片的图像采集（即使用智能手机摄像头 ） |  |  |  |  |  |

20. 请说明您用来收集患者**客观**信息任何其他方法或工具（如果有请注明，如果没有请填“无”）：

________________________________________________________________

21. 我们邀请您就常规临床评估信息的收集方法提出进一步的意见或建议。您的想法对我们很重要。

________________________________________________________________

**这部分是关于信息记录的：**

22. 请选择最佳答案，说明您使用所列方法或工具**记录**患者相关临床评估信息的**频率**

| 问题 | 从不使用 | 很少使用 | 有时使用 | 经常使用 | 总是使用 |
| --- | --- | --- | --- | --- | --- |
| 我用纸质版文字形式记录患者的康复信息 |  |  |  |  |  |
| 我使用结构化表格/模板形式记录患者信息 |  |  |  |  |  |
| 我使用非结构化自由文本形式将患者信息输入电子病历（EMR）系统 |  |  |  |  |  |
| 我在电子病历中使用结构化输入对笔记进行编码；例如标准化代码或下拉菜单(例如使用术语,如ICD-10 , ICF编码等) |  |  |  |  |  |
| 我和/或我的患者在共享的电子健康记录（如 “电子病历系统”）中进行记录 |  |  |  |  |  |
| 我将笔记输入到标准的文字处理或电子表格软件中（使用非结构化的自由文本，例如 Word 或 Excel） |  |  |  |  |  |
| 我使用标准的文字处理或电子表格软件中的预设格式或模板输入笔记 |  |  |  |  |  |
| 我将患者数据输入移动应用程序或专为医疗保健管理设计的在线平台，例如医疗健康管理应用，如：好大夫在线、平安好医生、春雨医生等 |  |  |  |  |  |
| 我的患者将自己的信息输入与我共享的移动应用程序或在线平台，如：好大夫在线、平安好医生、春雨医生等 |  |  |  |  |  |
| 我对患者信息进行录音（例如，通过录音机或录音软件） |  |  |  |  |  |
| 我以数字方式（例如使用设备或云储存）保存记录评估的图像和视频 |  |  |  |  |  |
| 我使用腾讯会议或类似的平台进行视频远程会诊 |  |  |  |  |  |

23. 请说明您用于**记录**临床信息的任何其他方法或工具（如果有请注明，如果没有请填“无”）：

________________________________________________________________

24. 请在此对您的常规临床评估信息记录做法提出任何最后意见？

________________________________________________________________

**第3部分 - 使用数字健康技术的意愿**

数字技术（例如智能手机、应用程序、电子健康记录、可穿戴传感器、数字视频等）可以用来支持医疗保健中的一系列不同功能。我们有兴趣了解**您是否愿意**使用数字健康技术来支持您作为康复治疗师的临床工作。

以下项目是世界卫生组织数字健康干预分类v1.0中的项目的改编。

25. 对于下面列出的每项功能，**您**有**多大意愿**使用数字技术来支持您的临床工作？

| 问题 | 完全不愿意 | 一点点 | 有点 | 相当多 | 非常愿意 |
| --- | --- | --- | --- | --- | --- |
| 核实患者的个人详细信息（例如新患者登记） |  |  |  |  |  |
| 预约门诊 |  |  |  |  |  |
| 跟踪患者的康复进展和/或临床服务使用情况 |  |  |  |  |  |
| 记录并输入患者的详细临床进展报告 |  |  |  |  |  |
| 使用标准化编码、复选框和下拉菜单记录或标记患者康复情况 |  |  |  |  |  |
| 记录和/或标记患者康复功能变化的指标 |  |  |  |  |  |
| 使用支持临床决策的软件促进我的思考 |  |  |  |  |  |
| 为我提供一份可供遵循的临床操作规程的数字化清单 |  |  |  |  |  |
| 对患者进行病情筛查 |  |  |  |  |  |
| 进行远程会诊 |  |  |  |  |  |
| 远程监控或追踪患者康复进展 |  |  |  |  |  |
| 向我发送有关患者病情的数据 |  |  |  |  |  |
| 与其他临床医生进行病例咨询/会诊/探讨 |  |  |  |  |  |
| 与科室上级进行信息汇报 |  |  |  |  |  |
| 就我的临床表现向我提供反馈 |  |  |  |  |  |
| 向我发送日常工作的更新以及工作流程通知 |  |  |  |  |  |
| 向我发送有关患者的非例行或突发健康事件警报 |  |  |  |  |  |
| 临床工作者同行的在线交流群组 |  |  |  |  |  |
| 为患者协调紧急响应和/或运输 |  |  |  |  |  |
| 管理医疗服务转诊或报告，例如向其他临床医生报告 |  |  |  |  |  |
| 管理向外部机构（如工作安全局等政府服务机构）的转介或报告 |  |  |  |  |  |
| 识别需要医疗服务的患者 |  |  |  |  |  |
| 安排我的临床活动 |  |  |  |  |  |
| 为我提供培训或教育内容 |  |  |  |  |  |
| 评估我的临床能力或表现 |  |  |  |  |  |
| 跟踪和管理患者的治疗处方 |  |  |  |  |  |
| 跟踪患者用药消耗情况 |  |  |  |  |  |
| 报告不良用药事件 |  |  |  |  |  |
| 向我发送影像诊断结果（如X线、CT、MRI等） |  |  |  |  |  |
| 记录并更新影像学检查的进度 |  |  |  |  |  |
| 从数字设备中获取诊断结果 |  |  |  |  |  |
| 跟踪病理情况（如血液化验） |  |  |  |  |  |

26. 您认为数字医疗技术还能帮助实现上表中未列出的其他**功能**吗？（请在下面注明）

________________________________________________________________

27. 请在此空白处就“**数字健康技术如何**在临床实践中为您提供康复治疗师支持”提出您的其他意见。

________________________________________________________________

恭喜您！您已完成我们的调查。感谢您的参与和宝贵意见，您的回答将为研究团队提供宝贵的见解。
