# Supplementary material for: Digital Health Technology Use Among Rehabilitation Professionals in China: Multi-Province Cross-Sectional Survey
Source: J Med Internet Res. 2026 Apr 9;28:e90623. doi: 10.2196/90623 (PMC13107106; doi:10.2196/90623)
Supplement: Multimedia Appendix 3 [file jmir_v28i1e90623_app3.docx]

**Multimedia Appendix 3. Survey Instrument for Rehabilitation Therapists on Digital Health Applications (Chinese back-translation to English for review)**

**Please confirm your eligibility to proceed with the survey**

1. Have you passed the National Health Professional Qualification Examination organized by the National Health Commission (NHC) and the Ministry of Human Resources and Social Security (MOHRSS), obtained a rehabilitation therapy qualification certificate, and are currently providing clinical rehabilitation therapy services in China?

□ Yes

□ No

2. In the past 6 months, have you treated or managed an average of at least 5 rehabilitation patients per week?

□ Yes

□ No

**Section 1 – Basic Information**

We would like to ask some brief questions about you and your clinical experience.

3. What is your professional title?

□ Rehabilitation Therapist Assistant

□ Rehabilitation Therapist

□ Senior Rehabilitation Therapist

□ Deputy Chief Rehabilitation Therapist

□ Chief Rehabilitation Therapist

□ Other (please specify): ____________________________________________

4. How did you learn about this survey?

□ Email

□ Social media (including WeChat Moments)

□ University/hospital website

□ Posters/flyers

□ Newsletters

□ Recommendation by colleagues or peers

□ Other (please specify): ____________________________________________

5. What devices do you use to access the internet? (Select all that apply)

□ Mobile phone

□ Tablet (e.g., iPad)

□ Desktop computer

□ Laptop

□ Smart TV

□ Gaming console

□ Smartwatch (e.g., Apple Watch, Huawei Watch, Xiaomi Band)

□ E-book reader (e.g., Kindle, Xiaomi Duokan)

□ Smart home assistant (e.g., Xiaomi AI, Tmall Genie)

□ Other (please specify): ____________________________________________

6. How often do you access the internet?

□ Hourly

□ Daily

□ Weekly

□ Monthly

□ Less than monthly

7. What is your gender?

□ Male

□ Female

8. What is your age range?

□ < 20

□ 20–29

□ 30–39

□ 40–49

□ 50–59

□ 60–69

□ 70+

9. How many years of clinical experience do you have (since obtaining your rehabilitation therapy qualification)?

□ < 2 years

□ 2–5 years

□ 6–10 years

□ 11–15 years

□ 16–20 years

□ > 20 years

10. What is your highest level of education?

□ Secondary school

□ College diploma

□ Bachelor's degree

□ Master's degree

□ Doctoral degree

□ Other (please specify): ____________________________________________

11. What type of institution/department do you work in for rehabilitation therapy?

□ Private institution

□ Public institution

□ Both private and public institutions

□ Other (please specify): ____________________________________________

12. What is the primary type of healthcare institution where you work? (Select your main workplace)

□ Rehabilitation department in a private hospital

□ Rehabilitation department in a public hospital

□ Rehabilitation specialty hospital

□ Community healthcare center

□ Elderly care facility

□ Disability rehabilitation center

□ Sports team/sports medicine institution

□ Other (please specify): ____________________________________________

13. On average, how many hours per week do you spend on clinical rehabilitation practice?

□ < 5 hours

□ 6–10 hours

□ 11–20 hours

□ 21–30 hours

□ 31–40 hours

□ > 40 hours

14. What is the city of your primary workplace?

15. What is your primary field of rehabilitation therapy practice?

□ Neurological rehabilitation

□ Musculoskeletal rehabilitation

□ Cardiopulmonary rehabilitation

□ Pediatric rehabilitation

□ Geriatric rehabilitation

□ Trauma rehabilitation

□ Oncological rehabilitation

□ Sports injury rehabilitation

□ Mental and psychological rehabilitation

□ Speech, swallowing, and hearing rehabilitation

□ Vocational rehabilitation

□ Other (please specify): ____________________________________________

16. Which of the following best describes your professional duties?

□ **Physical Therapist:** I use exercise, manual therapy, or physical agents instruments to treat patients' functional disorders, such as post-surgery rehabilitation or functional recovery after a stroke.

□ **Exercise Rehabilitation Specialist:** I specialize in designing exercise programs to help patients prevent sports injuries or enhance physical abilities, such as recovery training during the rehabilitation period.

□ **Occupational Therapist:** I assist patients in regaining daily living skills, such as relearning dressing, eating, or job-related skills.

□ **Speech Therapist:** I assess and improve patients' speech and swallowing disorders.

□ **Prosthetist & Orthotist:** I improve patients' quality of life and mobility through adjustments to prosthetics or orthotics.

□ **Cardio-Pulmonary Rehabilitation Therapist:** I specialise in designing and implementing rehabilitation programmes for patients with cardiopulmonary conditions, with the aim of enhancing their cardiopulmonary function and overall health.

□ **Pediatric Rehabilitation Therapist:** I evaluate and provide personalized therapy for children with speech disorders, developmental delays, autism, or cerebral palsy, improving their functions.

□ Other (please specify): ____________________________________________

**Section 2 – Assessment Methods**

The following questions pertain to how you subjectively and objectively collect and record patient information during rehabilitation therapy.

**Subjective Information Collection (information reported by patients):**

17. Indicate how frequently you use the following methods or tools to collect subjective clinical information from your rehabilitation patients.

| **Question** | **Never** | **Rarely** | **Sometimes** | **Often** | **Always** |
| --- | --- | --- | --- | --- | --- |
| Face-to-face interviews |  |  |  |  |  |
| Telephone interviews |  |  |  |  |  |
| Via electronic messages (e.g., Wechat, text, social media) |  |  |  |  |  |
| Via email (including attachments) |  |  |  |  |  |
| Through video conferencing/teleconsultation (e.g., Tencent Video, DingTalk) |  |  |  |  |  |
| Using paper questionnaires or outcome measures filled by patients |  |  |  |  |  |
| Using electronic systems to collect patient-reported subjective information (e.g., Word, Excel) |  |  |  |  |  |
| Patients upload information to shared electronic health records I can access (e.g., personal information entered via hospital apps during registration) |  |  |  |  |  |
| Patients use apps to log/track their condition, which I can access/view (e.g., apps for symptom monitoring or exercise prescriptions) |  |  |  |  |  |

18. Please specify any other methods or tools you use to collect subjective information from patients (if applicable):

________________________________________________________________

**Objective Information Collection (externally measured or examined information):**

19. Indicate how frequently you use the following methods or tools to collect objective clinical information from patients.

| **Question** | **Never** | **Rarely** | **Sometimes** | **Often** | **Always** |
| --- | --- | --- | --- | --- | --- |
| Visually assessing patients’ movements and functions |  |  |  |  |  |
| Evaluating patients’ movements and functions through video consultations (e.g., Tencent Meetings, DingTalk) |  |  |  |  |  |
| Using measurement tools (e.g., goniometers, measuring tape, grip strength meters, pressure cuffs) |  |  |  |  |  |
| Using sensors or devices to measure motion, posture, balance, gait, muscle activity (e.g., balance testers, gait analysis systems) |  |  |  |  |  |
| Patients use clinical sensors or apps to monitor motion, posture, balance, gait, and muscle activity; even when outside clinical/rehabilitation settings, I can remotely access this data (e.g., wearable posture sensors) |  |  |  |  |  |
| Using data from personal wearable activity trackers (e.g., Apple Watch, Huawei Watch, Xiaomi Band) |  |  |  |  |  |
| Using video games and virtual reality technology (e.g., HTC VIVE, Oculus Rift, Sony PlayStation VR) as part of rehabilitation therapy to gather information on patient performance/progress |  |  |  |  |  |
| Using motion capture technology to analyze functional levels, involving markers attached to patients’ bodies (e.g., treadmill gait analysis systems) |  |  |  |  |  |
| Using markerless motion analysis tools (e.g., iPi Motion Capture, Coach’s Eye) for evaluations |  |  |  |  |  |
| Using photo-based image capture (e.g., smartphone cameras) |  |  |  |  |  |

20. Please specify any other methods or tools you use to collect objective information from patients (if applicable):

_______________________________________________________________

21. We invite you to provide additional comments or suggestions regarding the methods of collecting routine clinical assessment information. Your feedback is valuable.

________________________________________________________________

**Information recording**

22. Indicate how frequently you use the following methods or tools to record clinical assessment information of patients.

| Question | Never | Rarely | Sometimes | Often | Always |
| --- | --- | --- | --- | --- | --- |
| I record rehabilitation information using handwritten notes |  |  |  |  |  |
| I record patient information using structured forms/templates |  |  |  |  |  |
| I input unstructured free-text information into electronic medical records (EMR) systems |  |  |  |  |  |
| I use structured input (e.g., standardized codes, dropdown menus) to encode notes in EMR systems (e.g., terms like ICD-10, ICF codes) |  |  |  |  |  |
| I or my patients record information in shared electronic health records (e.g., EMR systems) |  |  |  |  |  |
| I input notes into standard word processing or spreadsheet software (e.g., Word, Excel, using free text) |  |  |  |  |  |
| I use pre-set formats or templates in standard word processing or spreadsheet software to input notes |  |  |  |  |  |
| I input patient data into mobile apps or online platforms designed for healthcare management (e.g., Hao Daifu Online, Ping An Good Doctor, Chunyu Doctor, Rehab Journey) |  |  |  |  |  |
| Patients input their information into shared mobile apps or online platforms (e.g., Hao Daifu Online, Ping An Good Doctor, Chunyu Doctor, Rehab Journey) |  |  |  |  |  |
| I record patient information using audio recording devices or apps |  |  |  |  |  |
| I digitally save recorded images and videos from assessments (e.g., using devices or cloud storage) |  |  |  |  |  |
| I conduct teleconsultations via platforms such as Tencent Meetings |  |  |  |  |  |

23. Please specify any other methods or tools you use to record clinical information (if applicable):

________________________________________________________________

24. Please share any final comments on your routine clinical assessment recording practices:

________________________________________________________________

**Section 3 – Willingness to Use Digital Health Technologies**

Digital technologies (e.g., smartphones, apps, electronic health records, wearable sensors, digital video) can support various healthcare functions. We are interested in understanding your willingness to use digital health technologies to support your clinical work as a rehabilitation therapist.

The following items are adapted from the WHO Digital Health Interventions Classification v1.0.

25. How willing are you to use digital technologies to support the following clinical tasks?

| **Tasks** | **Not at all** | **Slightly** | **Somewhat** | **Quite** | **Very** |
| --- | --- | --- | --- | --- | --- |
| Verifying patient details (e.g., new patient registration) |  |  |  |  |  |
| Scheduling outpatient appointments |  |  |  |  |  |
| Tracking patient rehabilitation progress and/or clinical service utilization |  |  |  |  |  |
| Recording and entering detailed clinical progress reports for patients |  |  |  |  |  |
| Recording or coding patient rehabilitation status using standardized codes, checkboxes, or dropdown menus |  |  |  |  |  |
| Documenting or tagging metrics for changes in patient rehabilitation functions |  |  |  |  |  |
| Using clinical decision-support software to aid my thinking |  |  |  |  |  |
| Providing me with a digital checklist of clinical protocols to follow |  |  |  |  |  |
| Screening patients for health conditions |  |  |  |  |  |
| Conducting teleconsultations |  |  |  |  |  |
| Monitoring or tracking patient rehabilitation progress remotely |  |  |  |  |  |
| Sending me data on patient conditions |  |  |  |  |  |
| Consulting/discussing cases with other clinicians |  |  |  |  |  |
| Reporting information to department supervisors |  |  |  |  |  |
| Providing me with feedback on my clinical performance |  |  |  |  |  |
| Sending me notifications relevant to my daily work updates |  |  |  |  |  |
| Sending me alerts about non-routine or urgent patient health events |  |  |  |  |  |
| Joining an online discussion group with other clinical workers |  |  |  |  |  |
| Coordinating emergency responses and/or transport for a patient |  |  |  |  |  |
| Managing medical service referrals or reports, such as to other clinicians |  |  |  |  |  |
| Managing referrals or reports to external agencies (e.g., occupational safety bureaus) |  |  |  |  |  |
| Identifying patients who need medical services |  |  |  |  |  |
| Scheduling my clinical activities |  |  |  |  |  |
| Providing me with training or educational content |  |  |  |  |  |
| Assessing my clinical skills or performance |  |  |  |  |  |
| Tracking and managing patients’ treatment prescriptions |  |  |  |  |  |
| Monitoring patients’ medication consumption |  |  |  |  |  |
| Reporting adverse medication events |  |  |  |  |  |
| Sending me imaging diagnosis results (e.g., X-ray, CT, MRI) |  |  |  |  |  |
| Recording and updating imaging examination progress |  |  |  |  |  |
| Accessing diagnostic results from digital devices |  |  |  |  |  |
| Tracking pathology results (e.g., blood tests) |  |  |  |  |  |

26. Do you think digital health technologies could support tasks not listed above? (Please specify):

________________________________________________________________

27. Please share any additional comments on how digital health technologies could support your clinical work as a rehabilitation therapist.

________________________________________________________________

**Congratulations!**

You have completed the survey. Thank you for your participation and valuable input. Your responses will provide critical insights for the research team.
