# Supplementary material for: Digital Health Technology Use Among Rehabilitation Professionals in China: Multi-Province Cross-Sectional Survey
Source: J Med Internet Res. 2026 Apr 9;28:e90623. doi: 10.2196/90623 (PMC13107106; doi:10.2196/90623)
Supplement: Multimedia Appendix 4 [file jmir_v28i1e90623_app4.docx]

**Table S1.** Kruskal-Wallis Tests Comparing Digital Health Technology Use by Educational Level

| Outcome Variable | H | df | p | ε² | Post-hoc Comparisons (Bonferroni-adjusted p) |
| --- | --- | --- | --- | --- | --- |
| Electronic systems(Word, Excel, etc) | 10.823 | 3 | .013 | 0.024 | Associate vs Doctoral: .026 |
| Patients use apps to log/track | 5.679 | 3 | .128 | 0.008 | None |
| Sensor-based assessment | 24.801 | 3 | **<.001** | 0.068 | Associate vs Master (<.001)  Bachelor vs Master (.001) |
| digital device devices to transmit movement data | 13.695 | 3 | **.003** | 0.033 | Associate vs Master (.044)  Bachelor vs Master (.028) |
| Marker-based Motion capture | 23.052 | 3 | **<.001** | 0.063 | Associate vs Master (<.001)  Bachelor vs Master (<.001) |
| Free-text input into EMR | 12.214 | 3 | **.007** | 0.029 | Bachelor vs Master (.013) |
| Structured coding (e.g.,ICD-10/ICF) | 7.258 | 3 | .064 | 0.013 | None |
| Verify patient information (willingness) | 7.945 | 3 | .047 | 0.016 | None |
| Tracking patient rehabilitation progress (willingness) | 6.990 | 3 | .072 | 0.012 | None |
| Conduct teleconsultation (willingness) | 7.508 | 3 | .057 | 0.014 | None |

Note: ε² (epsilon squared) effect size was calculated as (H – k + 1) / (N – k), where k = number of groups (4) and N = total sample size (324). Interpretation: 0.01 = small, 0.06 = medium, 0.14 = large. Post-hoc pairwise comparisons were performed using Dunn's test with Bonferroni correction; only statistically significant adjusted p-values (p < .05) are reported.

**Table S2.** Kruskal-Wallis Tests Comparing Digital Health Technology Use by Professional Title

| Outcome Variable | H | df | p | ε² | Post-hoc Comparisons (Bonferroni-adjusted p) |
| --- | --- | --- | --- | --- | --- |
| Electronic systems(Word, Excel, etc) | 1.193 | 4 | .879 | 0.000 | None |
| Patients use apps to log/track | 3.481 | 4 | .481 | 0.000 | None |
| Sensor-based assessment | 18.904 | 4 | **<.001** | 0.047 | Rehabilitation Assistant vs Senior Therapist (.033)  Rehabilitation Therapist vs Senior Therapist (.016) |
| digital device devices to transmit movement data | 13.142 | 4 | .011 | 0.029 | None |
| Marker-based Motion capture | 9.445 | 4 | .051 | 0.017 | None |
| Free-text input into EMR | 11.851 | 4 | **.018** | 0.025 | Rehabilitation Assistant vs Associate Chief (.015)  Senior Therapist vs Associate Chief (.016)  Rehabilitation Therapist vs Associate Chief (.044) |
| Structured coding (e.g.,ICD-10/ICF) | 5.589 | 4 | .232 | 0.005 | None |
| Verify patient information (willingness) | 6.692 | 4 | .153 | 0.008 | None |
| Tracking patient rehabilitation progress (willingness) | 7.370 | 4 | .118 | 0.011 | None |
| Conduct teleconsultation (willingness) | 11.802 | 4 | .019 | 0.024 | Rehabilitation Assistant vs Senior Therapist (.015) |

Note: Data were analyzed using Kruskal-Wallis tests with post-hoc pairwise comparisons performed using Dunn's test and Bonferroni correction. Effect size ε² (epsilon squared) was calculated as (H – k + 1) / (N – k), where k = number of groups (5) and N = total sample size (324). Interpretation: 0.01 = small, 0.06 = medium, 0.14 = large. For outcomes with overall p > .05 or H < k, ε² is reported as 0.000, indicating negligible differences. Only statistically significant Bonferroni-adjusted p-values (p < .05) are shown in the post-hoc comparisons column.
